# Mayaro: an emerging viral threat?

**DOI:** 10.1038/s41426-018-0163-5

**Published:** 2018-09-26

**Authors:** Yeny Acosta-Ampudia, Diana M. Monsalve, Yhojan Rodríguez, Yovana Pacheco, Juan-Manuel Anaya, Carolina Ramírez-Santana

**Affiliations:** Center for Autoimmune Diseases Research (CREA), School of Medicine and Health Sciences, Universidad del Rosario, Bogotá, 111221 Colombia

## Abstract

Mayaro virus (MAYV), an enveloped RNA virus, belongs to the Togaviridae family and *Alphavirus* genus. This arthropod-borne virus (Arbovirus) is similar to Chikungunya (CHIKV), Dengue (DENV), and Zika virus (ZIKV). The term “ChikDenMaZika syndrome” has been coined for clinically suspected arboviruses, which have arisen as a consequence of the high viral burden, viral co-infection, and co-circulation in South America. In most cases, MAYV disease is nonspecific, mild, and self-limited. Fever, arthralgia, and maculopapular rash are among the most common symptoms described, being largely indistinguishable from those caused by other arboviruses. However, severe manifestations of the infection have been reported, such as chronic polyarthritis, neurological complications, hemorrhage, myocarditis, and even death. Currently, there are no specific commercial tools for the diagnosis of MAYV, and the use of serological methods can be affected by cross-reactivity and the window period. A diagnosis based on clinical and epidemiological data alone is still premature. Therefore, new entomological research is warranted, and new highly specific molecular diagnostic methods should be developed. This comprehensive review is intended to encourage public health authorities and scientific communities to actively work on diagnosing, preventing, and treating MAYV infection.

## Introduction

Several tropical diseases, including some caused by arboviruses, are considered by the World Health Organization to be “neglected” and remain a continuing public health challenge^1,2^. Mayaro virus (MAYV) is an arbovirus and part of the Semliki complex that consists of seven other viruses: Bebaru, Chikungunya (CHIKV), Getah, Semliki Forest, Ross River (RRV), O’nyong-nyong, and Una viruses. This complex forms a serological group within the *Alphavirus* genus that shares some common antigenic sites, thus generating cross-reactivity with polyclonal immune sera among the species. The Semliki complex usually causes human disease characterized by fever, arthritis, and skin rash^3^. MAYV cases in humans have been limited to Central and South America, particularly to regions in and around the Amazon basin (i.e., the Amazon River and its tributaries)^4^. Evidence accumulated over time shows that when a virus is introduced into new environments, new species of mosquitoes might be involved in the transmission cycle^2,5^. On the other hand, both CHIKV and Zika virus (ZIKV) infections emerged in the Americas and rapidly spread to dozens of countries, affecting millions of people from 2013–2016. These countries could now become high-risk areas for MAYV infection, which may likely be misdiagnosed as CHIKV infection due to their similarities^6^.

## Biology of Mayaro virus

### Genomic organization and viral structure

**Fig. 1:**
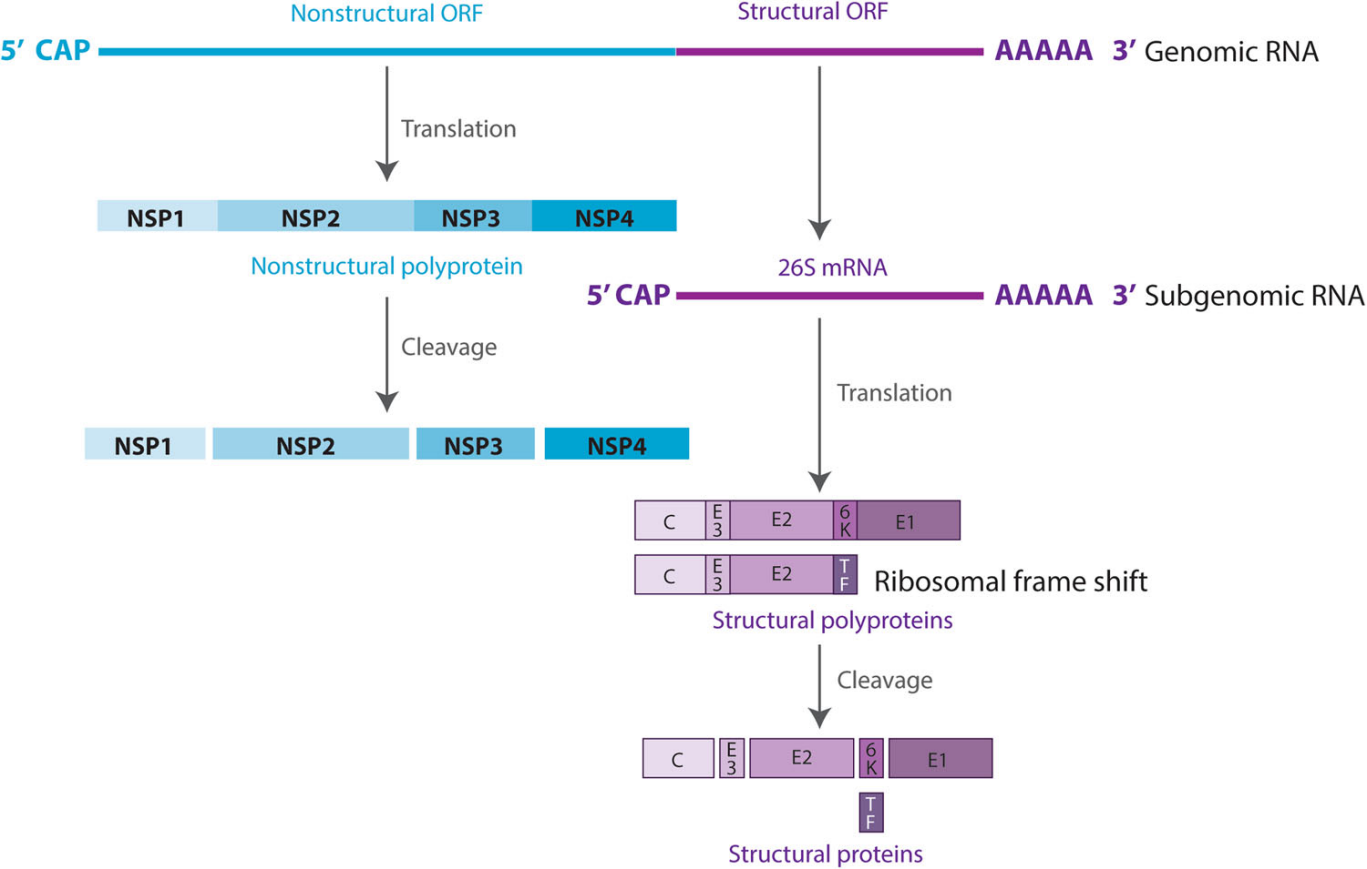
Schematic diagram of the MAYV genome and proteins. RNA is shown as a line and viral proteins are shown as boxes

The MAYV genome is composed of a positive-strand RNA of ~11.5 kb containing two open reading frames (ORFs). The 5′-proximal ORF encodes the nonstructural proteins, whereas the 3′-proximal ORF encodes the structural proteins. The nonstructural proteins are directly translated from the genomic RNA into one polyprotein, which is cleaved into four nonstructural peptides (nsP1, 2, 3, and 4). The structural proteins are translated from a subgenomic mRNA (26S mRNA), thus encoding a polyprotein cleaved into six proteins: capsid (C), envelope (E) E1, E2, E3, 6K, and transframe (Fig. 1)^7^.

The MAYV life cycle starts with binding of the viral envelope with an unknown cellular receptor, followed by endocytosis of the virus into the cytoplasm^8^. After the virus enters the host cell, disassembly of the core and release of genomic RNA occur. Once in the cytoplasm, genomic RNA is translated into nonstructural proteins. These proteins then enable the processing of genomic RNA into subgenomic mRNA and further translation into structural proteins. These are then processed and assembled into a nucleocapsid and glycoproteins, which, in association with the plasma membrane, result in the budding of the new virion particles^7^.

### Phylogenetic studies

Phylogenetic studies using whole-genome sequencing have classified MAYV strains into three genotypes: (1) genotype D, largely distributed in South America and the Caribbean; (2) genotype L, limited to North-central region of Brazil, and (3) genotype N, a newly described clade found only in a localized region in Peru^9^. Phylogenetic analyses and nucleotide sequence homologies confirm that MAYV belongs to the Semliki Forest complex. Moreover, analyses on the E1 region have shown that MAYV is related to the Una virus, the only other South American virus associated with Old World viruses^10^. Based on these results and the *Alphavirus*’ diversity and pathogenicity, it has been suggested that alphaviruses may have an Old World origin. The genotype L seems to be restricted to Brazil, suggesting a geographic constraint on MAYV dispersal. The fact that genotype L has not been found in other countries suggests a sampling bias rather than true viral strain subdivision^9^.

### Cellular and molecular mechanisms of pathogenicity

Most of the knowledge regarding the cellular and molecular mechanisms involved in alphavirus-induced arthritis comes from studies of RRV and CHIKV. Alphaviruses seem to disseminate through the host via lymphatics and microvasculature after subcutaneous inoculation by a mosquito bite^11^. The blood carries most viruses, as free virions or in the form of infected monocytes, to target organs. The liver and spleen, in turn, are the sites where further viral replication occurs, contributing to virus dissemination. Then, the virus reaches the bones, muscles, and articular tissues, and generates the acute phase of the disease, which is strongly associated with a local inflammatory process^12^. An inflammatory infiltrate rich in monocytes, macrophages, natural killer cells, and CD4^+^ and CD8^+^ T lymphocytes, affecting the joints and muscles, has been reported in murine models^13–15^.

The in vitro study by Cavalheiro et al.^16^ showed that MAYV infection of macrophages leads to apoptosis. MAYV replication in macrophages induces tumor necrosis factor (TNF) synthesis in association with fever, since TNF promotes an inflammatory profile characteristic of arthritis. Additionally, they observed an increase of reactive oxygen species (ROS) at early points of infection, which coincided with the acute phase of viral replication followed by TNF secretion^16^. These findings were confirmed by Camini et al.^17^, who found that MAYV induces significant oxidative stress in infected HepG2 cells. An imbalance in the production of ROS and the cell’s inability to detoxify these reactive species may be responsible for this status^17^.

Furthermore, Santiago et al.^18^ demonstrated that individuals with confirmed MAYV infection elicit a strong immune response, resulting in the secretion of pro-inflammatory immune mediators. During the acute phase of infection, various pro-inflammatory innate immune factors become active, such as interleukin (IL)-6, IL-7, IL-8, IL-12p70, IL-15, IP-10, and MCP-1. The chemokine MCP-1, which controls the migration and infiltration of monocytes and macrophages, is higher during the MAYV acute phase and persists at elevated levels for up to 6 months after infection. IL-2 and IL-9 are involved in cell proliferation and are also present at high levels during the convalescent phase, whereas IL-7 and IL-13 remain elevated until 3 months post infection. During the chronic phase, infected patients showed increased levels of IL-1β, IL-5, IL-10, IL-12p70, IL-17, interferon (IFN)-γ, and TNF-α. Other immune mediators showed higher levels in the blood compared to healthy donors (e.g., IL-1Ra, IL-6, IL-7, IL-8, IL-13, IL-17, G-CSF, IFN-γ, PDGF-BB, TNF-α, VEGF, and IL-12p70) regardless of the phase of MAYV infection. The profile of these inflammatory mediators has been associated with the severity and persistence of infection. Furthermore, diverse chemokines remained elevated in patients that developed persistent arthralgia: G-CSF, IL-1Ra, IL-8, IL-17, IFN-γ, MCP-1, PDGF-BB, and TNF-α. VEGF was also significantly elevated 3 to 6 months post infection. Based on these findings, Santiago et al.^18^ suggested that, similar to CHIKV, the MAYV immune response is mainly inflammatory during the acute phase. However, the composition of elicited immune mediators is distinct.

## Epidemiology

**Fig. 2:**
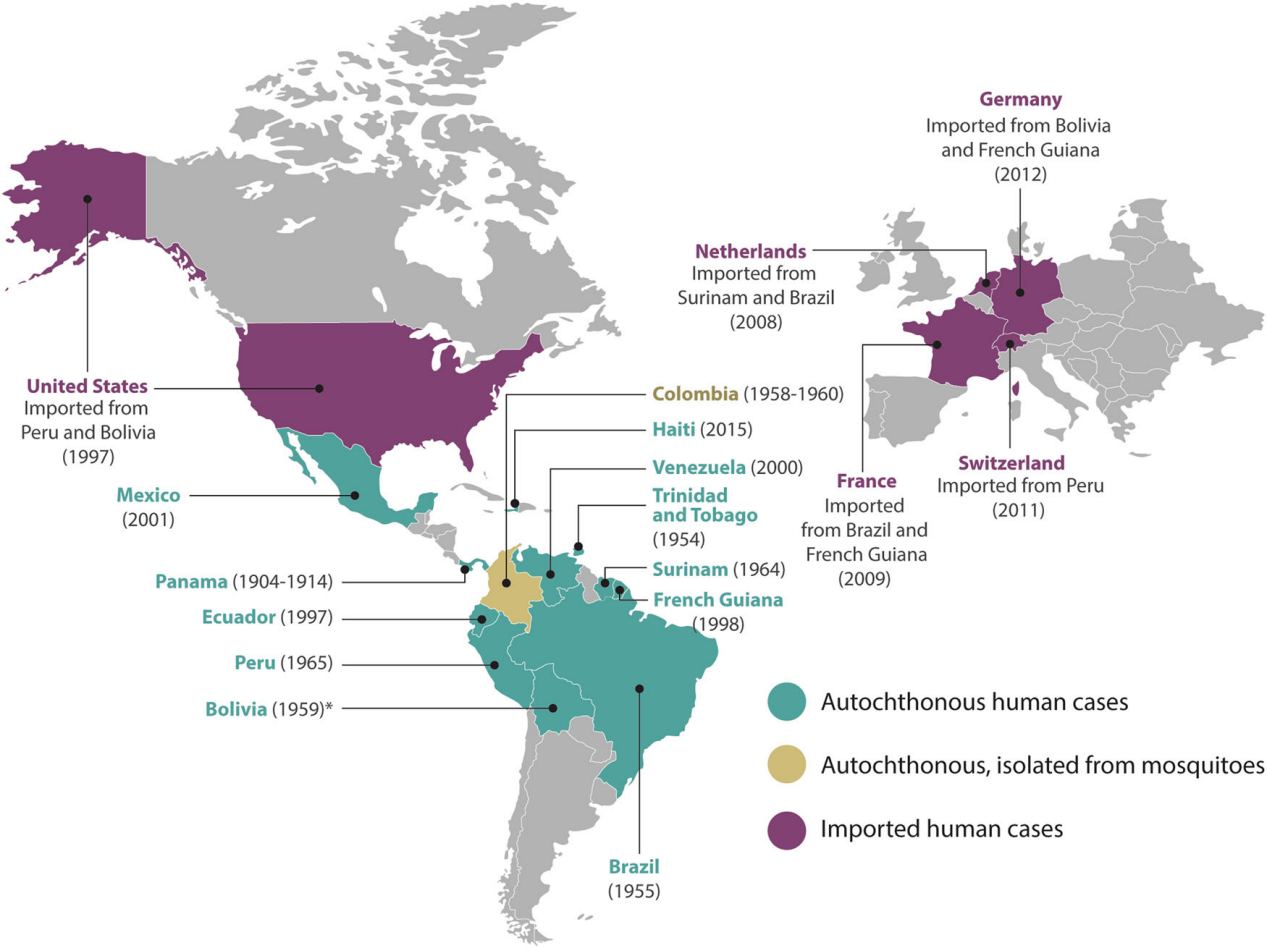
Epidemiological distribution of MAYV cases around the world. The year corresponds to the first reported case in the country. *The year of this report corresponds to the date of the article

Most of the epidemiology data available are based on serological tests, which may be highly cross-reactive with other alphaviruses. MAYV was first isolated in Trinidad and Tobago from the blood of five symptomatic infected humans during August and September of 1954. Four of the five cases were male forest workers, whereas the fifth was a female urban resident (Fig. 2)^19^. Nevertheless, in a retrospective study, MAYV was evidenced in sera collected during the construction of canals in Panama and Colombia^20^, which took place between 1904 and 1914. Chronologically, a MAYV fever epidemic occurred in Belém, Brazil in April of 1955. From this outbreak, six strains of MAYV were isolated from six patients. On the other hand, 74 patients from the same geographical area were tested for neutralizing antibodies against Semliki virus, of which 18.9% were positive. Since Semliki virus is confined to Africa, the authors assumed that all the Semliki-positive sera at that time represented MAYV infection^21^. In Bolivia, on the border with Brazil, a MAYV strain previously known as Uruma was possibly responsible for up to 15% of the “jungle fevers” among Okinawan colonists^22^. Moreover, between 1958 and 1960, 12 strains of arboviruses were isolated from forest mosquitoes collected in Santander, Colombia and included four strains of MAYV, all from *Psorophora* mosquitoes^23^. In 1964, a febrile illness was reported among Dutch soldiers in Surinam and was quite possibly due to infection with MAYV^24^. One year later, in 1965, MAYV was identified in Peruvian sera by neutralization tests carried out in Vero cell cultures^25^. In 1977, there was a MAYV outbreak in Pará, Brazil, where ~20% of the >4000 inhabitants were infected, and a very high proportion of those infected suffered overt clinical illness^26^.

In 1997, an outbreak of hemorrhagic fever was reported in one of the Amazonian military bases of Ecuador. The study showed that seroprevalence of MAYV infection was 20 times higher in Amazonian natives (46%) in comparison with individuals from other areas (2%). The results provided the first evidence of the presence of MAYV infection in Ecuador and a first assessment of risk factors for the spread of this *Alphavirus*^27^. In 1998, MAYV was reported for the first time in French Guiana and exhibited 6.3% seroprevalence. This study indicated that MAYV seroprevalence rates increased with age and with proximity to a tropical rainforest^28^.

The first human cases of MAYV in Venezuela occurred in January 2000 in the State of Miranda in four adult members of the same family. Serological results obtained 3 months after the onset of symptoms showed high specific MAYV IgM and IgG antibody titers^29^. In January 2010, 77 cases were recorded in the State of Portuguesa, Venezuela, representing one of the largest outbreaks of MAYV detected in South America. MAYV was isolated from acute-phase serum samples from 6 symptomatic patients^9^. Between May 2000 and December 2007, MAYV isolates were detected in rural areas of Bolivia and Peru^30^. Due to a follow-up study done at the Mexican Institute of Social Security in 2001, IgM MAYV antibodies were detected in 2 of 35 patients with hemorrhagic fever. One of these patients presented hemorrhages, thrombocytopenia, jaundice, and encephalopathy that led to death^31^.

In a surveillance study from 2010 to 2013 in 15 health centers in 4 Peruvian cities (Iquitos, Yurimaguas, Chanchamayo, and Puerto Maldonado), a 0.8% incidence of MAYV was detected. This study showed that 11 of the 16 persons with fever had MAYV isolated by cell culture assays, 13 were positive by reverse transcription PCR (RT-PCR) specific for MAYV, and all 16 had IgM ELISA seroconversion within 20 days of the acute phase^32^.

Other serosurveys in Brazil between 1966 and 2010 recurrently identified traces of MAYV infection, thus showing that this country is among the most affected by MAYV. This effect may be explained by the fact that Brazil is a tropical country largely covered by rainforests and other natural ecosystems, which provide ideal conditions for many arboviruses^33^.

In 2015, MAYV was recovered from an 8-year-old boy with an acute febrile illness in a semirural area of Haiti. The child was diagnosed through genome sequencing with co-infection for DENV-1 and MAYV, making it possible to rule out CHIKV infection^34^. The fact that the MAYV infection was found in someone from a nonforest area and that it occurred in the context of a DENV co-infection suggests that *A. aegypti* may have been the mosquito vector responsible for transmission. Notably, wild nonhuman primates are not native to Haiti, which could suggest a different reservoir or human-to-human transmission by *Aedes* mosquitoes^6^.

In addition, cases in North America imported from Peru^35^ and Bolivia^36^ have been reported. In Europe, the first imported case was observed in the Netherlands in 2008 and came from Surinam^37^. Another case was imported to the Netherlands in 2013 from Brazil^38^. One year later, in 2009, a French traveler was diagnosed with MAYV infection after returning from Brazil^39^. Another case imported to France from French Guiana was reported in 2016^40^. In 2011, a Swiss tourist showed high titers of IgM and IgG MAYV antibodies after visiting northern Peru^41^. In 2012, a woman from Germany had a febrile illness after visiting Bolivia. Serological analysis demonstrated MAYV-specific IgM and IgG antibodies 2 months after the onset of symptoms^42^. In 2013, another case was imported to Germany from French Guiana. The serum sample had a MAYV load of 1.24 × 10^7^ copies per mL^43^.

Due to the extensive distribution of MAYV in the Americas (Fig. 2), MAYV fever may become a public health issue in the near future, thus imitating the epidemiological evolution of other arboviruses.

## Modes of transmission

Some outbreaks have been associated with rainy and summer seasons, when arthropods reproduce and the virus begins its cycle between the viral vector and its target. MAYV transmission occurs when female mosquitoes belonging to the genus *Haemagogus*, mainly *Haemagogus janthinomys*, acquire blood from an infected host with high concentrations of MAYV in its blood^44^. MAYV has been isolated from other genera of mosquitoes, including *Culex*, *Mansonia*, *Aedes*, *Psorophora*, and *Sabethes*^23^. Once the virus is ingested, it infects the epithelial cells of the midgut or mesenteron of the mosquito. The virus undergoes replication and migrates to the basal lamina to reach the hemolymph. Then, the virus invades the salivary glands, where it establishes a persistent infection. The virus is released through the saliva into the blood when the mosquito bites a potential host. An increased likelihood of spreading to other geographical locations is also facilitated by the extensive host elasticity of MAYV. MAYV has been detected in nature in several vertebrate hosts, such as nonhuman primates, rodents, birds, sloths, and other small mammals^45^.

The geographical distribution of MAYV vectors and hosts has restricted most outbreaks to rural areas close to tropical forests. However, several factors suggest the possibility of MAYV urbanization^46,47^: (1) its homology with CHIKV, another *Alphavirus* with well-described history of urbanization; (2) the regular occurrence of MAYV cases near the main tropical cities where *A. aegypti* is endemic^35^; (3) experimental studies showing that *A. aegypti*^6,48^ and *A. albopictus*^49^ are competent vectors for MAYV transmission; (4) the fact that MAYV is spread by sick travelers^39,42^ or migratory birds^50^. In addition, Junt et al.^51^ reported an accidental laboratory infection likely due to aerosol exposure. There are no data thus far regarding other types of transmission (e.g., vertical, sexual, or via other fluids, including saliva and tears).

## Clinical manifestations

The symptoms caused by MAYV are nonspecific, mild, and self-limited. Its clinical course is typically described as two phases: acute and subacute^52^. The acute phase is characterized by a short, transient viremia (3–4 days), followed by an incubation period of 7–12 days, in which the systemic symptoms become evident. Among the common symptoms described in the literature are a triad of abrupt fever, arthralgia/arthritis, and maculopapular rash often related to bleeding^42,53,54^. However, other symptoms can occur, such as headache, myalgia, retro-orbital pain, vomiting, and diarrhea. The fever can last 10 days and may reappear after a period free of hyperthermia. This pattern can help to distinguish fever caused by MAYV from other arboviruses^53^. MAYV causes an acute incapacitating disease, and >50% of cases can be followed by long-term arthralgia. Comparable to CHIKV fever, joint pain can persist for several months^18^. In addition, as with other *Alphavirus* infections, MAYV can produce severe complications, such as intermittent fever, neurological complications, myocarditis, and even death^55^. Hemorrhagic manifestations are rare but have been described by Mourao et al.^56^.

**Fig. 3:**
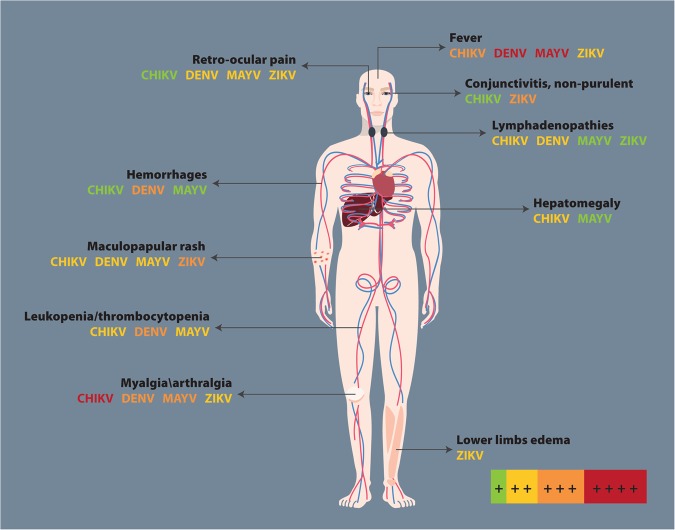
Clinical findings in CHIKV, DENV, MAYV, and ZIKV. Adapted from^57^. The figure includes a color code to indicate the symptom intensity produced by each arbovirus: red corresponds to ++++, orange to +++, yellow to ++, green to +, and the absence of the arbovirus name means no symptoms

When an infection due to arbovirus is suspected, the term “ChikDenMaZika syndrome” has been suggested to denote the common symptoms shared by CHIKV, DENV, MAYV, and ZIKV infections given the pattern of co-infection and co-circulation in South America^57^. Some of these arboviruses can cause hepatitis, lymphadenopathies, thrombocytopenia, and leukopenia (Fig. 3). Notably, there is a high probability of misdiagnosis, especially during early clinical stages, which constitutes a challenge^57^.

## Treatment

As is the case for other arboviruses, no specific treatment is available for MAYV infection, and only supportive care is offered to patients^32,58^, with the goal of treating pain and fever using analgesics and/or nonsteroidal anti-inflammatory drugs (NSAIDs)^47,58^. Several drugs used to treat symptoms associated with CHIKV infection may also be used to treat MAYV infection symptoms, leading to faster healing of joint pain and soft tissue manifestations^59^. Since nontherapeutic options are available, one possibility for CHIKV and MAYV fevers assessment could be passive immunization in order to prevent and treat the diseases while vaccine discovery still ongoing^47^.

Corticosteroids (prednisolone) have been administered in a few cases, but there is no evidence of their efficacy^54^. In some cases, concomitant use of steroids and NSAIDs or low-dose aspirin has been offered; however this is not recommended due to risk of upper gastrointestinal bleeding^60^. Antimalarial drugs (e.g., chloroquine) are an alternative for arthralgia associated with CHIKV and MAYV infection^61^.

## Diagnostic methods

MAYV is part of the Semliki complex, a serological group in the *Alphavirus* genus that shares some common antigenic sites, leading to cross-reactivity with polyclonal immune sera among the species. This situation may cause misdiagnoses and consequent underreporting of Mayaro fever in endemic areas of arboviruses^54^.

### Serological detection

**Fig. 4:**
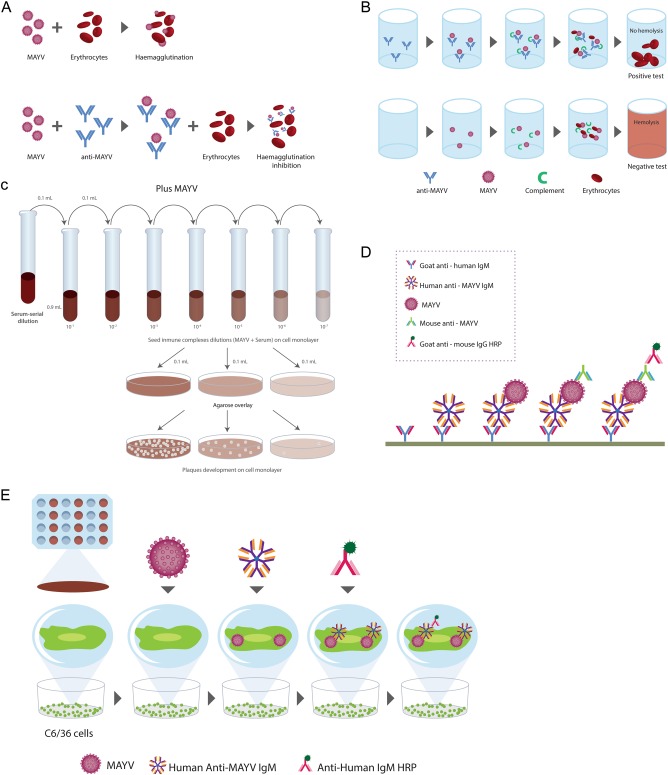
Serological tests for MAYV detection. **a** Hemagglutination inhibition. **b** Complement fixation. **c** Neutralization test. **d** IgM capture enzyme-linked immunosorbent assay. **e** Enzyme immunoassay using infected cells as antigens

Several serological detection techniques use virus-infected cells, recombinant antigens of structural or nonstructural proteins, or whole virus. Enzyme-immune assays can detect the serological response to MAYV infection. These immunoassays are rapid and sensitive at detecting and differentiating between IgM and IgG antibodies. IgM antibodies against MAYV appear 3 days after the onset of symptoms and persist for up to 3 months, whereas IgG antibodies persist for years^47,62^. To date, five serological tests have been used for the detection of MAYV: hemagglutination inhibition (HI), complement fixation (CF), neutralization test (NT), IgM capture enzyme-linked immunosorbent assay (MAC-ELISA), and enzyme immunoassay using infected cells as antigens (EIA-ICC) (Fig. 4). Serological methods include commercial kits and in-house assays. A combination of different methods should be used to reduce the risk of a misdiagnosis due to cross-reactivity among arboviruses.

The most frequent test used for routine serological diagnosis of DENV infections^63^, HI, is also used for the diagnosis of MAYV due to its low cost and simplicity. However, a disadvantage of HI is that it requires goose erythrocytes and a second blood sample 15 days after the first sample was collected in order to confirm the diagnosis^64^.

The CF test, which can identify anti-MAYV antibodies, is more specific. However, in comparison to the HI test, it is less sensitive, more difficult to set up and more expensive. This test uses complement consumption as a principle during the antigen-antibody reaction. The antibodies detected for CF generally appear later than HI antibodies and persist for shorter periods. They are, therefore of limited value for sero-epidemiological studies^64^.

MAYV produces cytopathic effects that can be observed and quantified as plaques in cell culture. Thus, it is easily exposed by the plaque reduction NT (PRNT), which measures antibodies that are able to neutralize and prevent the infection of Vero-76 or BHK-21 cells. The specificity and sensitivity of this test makes it the gold standard serological test for alphaviruses^65^. Although the PRNT provides the best specificity for MAYV and other arboviruses, all serological assays are subjected to cross-reactivity, especially in patients from endemic areas. For this reason, combinations of techniques are necessary to obtain a differential diagnosis^41^. To date, a MAYV diagnostic algorithm has not been established.

In contrast, Figueiredo et al.^66^ demonstrated that MAC-ELISA is a practical and valid technique for the detection of antibodies produced during the first few days of a primary MAYV infection. In addition, the diagnosis of MAYV infection can also be performed by IgG/IgM detection in EIA-ICC. This assay uses *A. albopictus* C6/36 cells cultured in Leibovitz L-15 medium^66^.

### Molecular detection

Molecular methodologies have become valuable tools to genetically typify viruses and provide insight into the nature of viral transmission cycles and epidemiological patterns. Most methods based on PCR, including nucleic acid sequence-based amplification assays and real-time RT-PCR, are excellent molecular diagnostic methods for *Alphavirus* detection. This is due to the high specificity and sensitivity in the early viremic stage of illness, since the target nucleic acids are generally present in the blood for only 2–6 days after infection. These techniques use generic primers for alphaviruses or specific primers for MAYV that amplify the *nsP1*, *nsP2*, or *E1* genes^67,68^. The primers anneal to a highly conserved sequence encoding the nsP1, nsP2, or E1 proteins, making it possible to amplify most genes from several highly divergent alphaviruses^69^. After this step, a nested PCR can be used for further confirmation of MAYV infection, although this method may introduce false positives^69^. Naveca et al.^70^ described a sensitive method for the simultaneous detection of MAYV and Oropouche by multiplexed one-step RT-qPCR. This technique is highly accurate and sensitive (>98% for both viruses), with a threshold of detection between 2 and 20 copies. This is a new tool for the differential diagnosis of MAYV in arbovirus-endemic areas^70^.

Wang et al.^71^ developed a rapid and simple method for diagnosing acute *Alphavirus* infection. This method combines the sensitivity of PCR, the simplicity of ELISA, and the specificities of sequence-specific DNA probes. After amplification using RT-PCR with primers targeting conserved sequences in the *nsP1* gene, sequence-specific, biotin-labeled probes targeted against MAYV genes are used for the detection of amplicons using ELISA^71^.

Given the concern about MAYV emergence in the Americas and the few molecular tests that have been evaluated in the literature, Waggoner et al.^72^ recently developed an rRT-PCR for MAYV to be used in human plasma and urine samples spiked with different MAYV strains. No detection was observed when the MAYV rRT-PCR was tested with genomic RNA from related arboviruses^72^.

## Prevention and control

The current strategy for controlling, preventing, and reducing the transmission of MAYV depends on interrupting human-vector contact and includes the following: (1) the application of insecticides at breeding sites; (2) the use of residual insecticide treatment of adult resting places; and (3) the utilization of natural enemies or competitors to reduce the vector density^58,73^. The isolation of viremic individuals to prevent transmission to local mosquitoes could also be appropriate in disease-free countries where functional vectors exist. Finally, immunization would be the best means of prevention, although there is no licensed vaccine against MAYV. However, Weise et al.^74^ developed a live-attenuated vaccine called MAYV/IRES, which is highly immunogenic, noninfectious to mosquito cells, and protective in murine models. These results indicate that further preclinical development of the MAYV/IRES vaccine is justified since MAYV can become an epidemic, such as the closely related CHIKV^74^.

Amorin et al.^75^ proved the antiviral effect of thienopyridine derivatives on MAYV replication. These affect early and late stages and morphogenesis, in particular showing the potential application of this new class of antiviral drugs with low cytotoxicity and good bioavailability^75^. In addition, a recent study published by Ferreira et al.^76^ showed a strong inhibitory effect of epicatechin on MAYV growth, indicating that this is a promising candidate against MAYV.

## Public health interventions

**Fig. 5:**
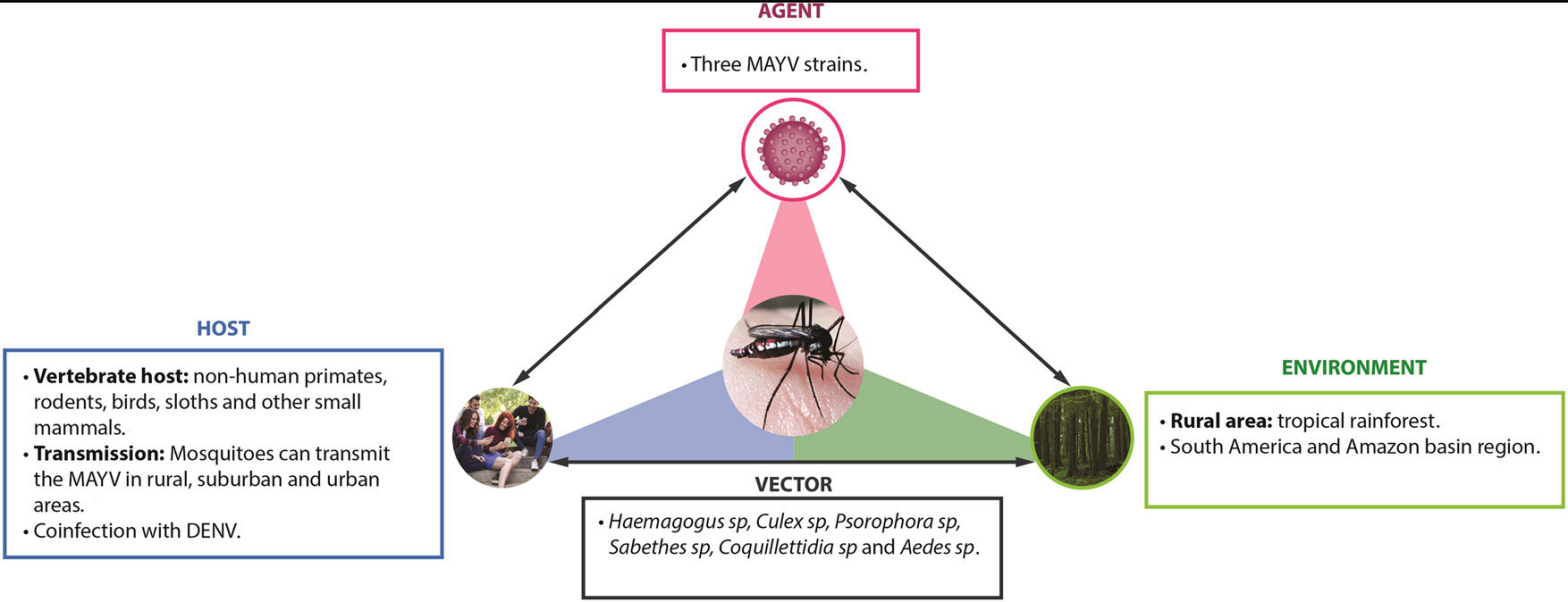
Public efforts on MAYV infection should focus not only on the vector, but on the host and its environmental surrounding. The Mayaro virus epidemiologic triad

Public health surveillance systems are important for the early identification of virus outbreaks. Once identified, it is essential for healthcare providers and public health officials that local health authorities establish a rapid and aggressive control response to mosquitoes^77^. Some countries have developed and implemented an early-warning system that can predict epidemics by arboviruses, identify high-risk zones for transmission and amplification of the agent, and acquire information on meteorological and spatial factors that could influence vector dynamics^78,79^. Beyond detecting the number of cases, it should be noted that travelers can potentially act as sentinels for emerging infectious diseases. MAYV infection should be considered in cases of patients who have recently visited tropical areas in South America. Regarding this finding, a partnership should be developed between public health surveillance systems and transportation companies to provide epidemiological control^42^. Digital participatory surveillance systems may be used to detect real-time incidence of symptoms compatible with arboviral diseases and other tropical imported diseases. Permanent epidemiological and entomological studies should be carried out to determine MAYV endemic areas and the risk of transmission to human hosts, especially in countries close to regions where the disease has already been confirmed. The interactions of the environment, including the vector, natural history of MAYV, and host, are reflected in Fig. 5.

## Conclusions

MAYV infection in humans has mainly been reported in rural areas, and few of these cases have been exported to urban areas. Nevertheless, given the latest pathogenic outbreaks of ZIKV, DENV, and CHIKV, it is important to carefully monitor MAYV evolution by implementing lessons learned from previous arbovirus outbreaks to avoid further public health issues. Indeed, the factors associated with arbovirus outbreaks include the following: (1) human modification of the ecosystem (e.g., agriculture, population growth, urbanization, human migration, and deforestation); (2) adaptation of viruses to different vectors by genetic mutations; (3) resistance of vectors to insecticides and resistance of pathogens to drugs; and (4) climate changes triggering the spread of vectors within a new geographic region where populations are immunologically naive, inducing epidemic bursts.

As discussed above, *Aedes* moesquitoes, specifically *A. aegypti*, are susceptible to MAYV infection and are potential candidates for the urban life cycle. The short time of viremia in humans could explain the low probability of transmission from humans to *A. aegypti* observed until now. However, genetic modifications of the virus could increase its infectivity to urban vectors. The genetic mutations together with environmental changes allow arboviruses to colonize and be transmitted by new vectors, such as *A. aegypti*. This has already been observed with yellow fever virus, DENV, and CHIKV. Therefore, it is probable that MAYV will develop the same pattern.

Moreover, to avoid assumptions from *Alphavirus* infections to MAYV, it is important to dissect the specific pathophysiologic mechanisms of this virus. In addition, research and implementation of specific and precise diagnostic methods are warranted to distinguish MAYV from other arboviral infections. Finally, medical training of MAYV clinical symptoms must be a priority for care providers in arbovirus-endemic areas, and the follow-up of these infected patients is necessary to evaluate complications.
